# A novel, sensitive dual-indicator cell line for detection and quantification of inducible, replication-competent latent HIV-1 from reservoir cells

**DOI:** 10.1038/s41598-019-55596-8

**Published:** 2019-12-18

**Authors:** Fanny Salasc, David W. Gludish, Isobel Jarvis, Saikat Boliar, Mark R. Wills, David G. Russell, Andrew M. L. Lever, Hoi-Ping Mok

**Affiliations:** 1 Department of Medicine, University of Cambridge, Cambridge, UK; 2 Cornell University College of Veterinary Medicine, New York, USA; 3 Yong Loo Lin School of Medicine National University of Singapore, Singapore, Singapore

**Keywords:** Microbiology techniques, Retrovirus

## Abstract

Understanding the mechanisms involved in HIV infection and latency, and development of a cure, rely on the availability of sensitive research tools such as indicator cells, which allow rigorous quantification of viral activity. Here we describe the construction and validation of a novel dual-indicator cell line, Sup-GGR, which offers two different readouts to quantify viral replication. A construct expressing both *Gaussia* luciferase and hrGFP in a Tat- and Rev-dependent manner was engineered into SupT1-CCR5 to create Sup-GGR cells. This cell line supports the replication of both X4 and R5-tropic HIV as efficiently as its parental cell line, SupT1-CCR5, and allows repeated sampling without the need to terminate the culture. Sup-GGR demonstrates comparable sensitivity and similar kinetics in virus outgrowth assays (VOA) to SupT1-CCR5 using clinical samples. However the *Gaussia* luciferase reporter is significantly less labor-intensive and allows earlier detection of reactivated latent viruses compared to the conventional HIV p24 ELISA assay. The Sup-GGR cell line constitutes a versatile new tool for HIV research and clinical trials.

## Introduction

Despite significant advances in our understanding of HIV infection and the development of effective antiretroviral therapy, HIV continues to drive global morbidity and mortality while a cure remains elusive. A major obstacle in the HIV cure effort is the persistence of latently infected reservoir cells even after prolonged anti-retroviral therapy. An important tool in the detection of this relatively small pool of reservoir cells is an indicator cell line. Indicator cells expressing different reporters to identify active viral replication have been invaluable in the detection and characterization of latently infected cells. Examples include Magi cells, a HeLa based cell line with an HIV-LTR driven beta-galactosidase^1^, and TZM-bl which, in addition to beta galactosidase, also have a luciferase reporter^2^. However, these and other existing systems suffer from their inability to permit repeated sampling of the same culture for viral activity, as cells must be lysed to analyze reporter readouts, thus limiting their utility. Here we report construction and characterization of a novel dual-indicator cell line, Sup-GGR (**G**aussia **G**FP **R**eporter) that overcomes this limitation. These cells are derived from SupT1-CCR5, a T-lymphoblastic lymphoma cell line that stably expresses the HIV-1 receptor CD4, and the co-receptors CCR5 and CXCR4, allowing entry of both X4 and R5 tropic viruses^3^. Sup-GGR contains a Tat/Rev dependent expression cassette^4^ that produces both humanized *Renilla* GFP (hrGFP) and *Gaussia* luciferase (GLuc) upon HIV infection^5^.

Uniquely, *Gaussia* luciferase is secreted into the growth media; as such these supernatants can be harvested for reporter readout and replaced with fresh media so that the same culture can be maintained for subsequent harvests at different time points.

We validated Sup-GGR cells in virus outgrowth assays (VOA) using clinical samples from HIV infected patients. VOA detects inducible, replication-competent HIV in a rigorously defined population of latently infected resting CD4 T cells, and is the gold standard in quantifying the replication competent latent reservoir. We had previously reported that using SupT1-CCR5 in VOA vastly improved the reproducibility of the assay^6^. Here we made a head to head comparison, and found that the novel Sup-GGR cell line is comparably efficient to SupT1-CCR5 in supporting the replication of a range of laboratory and clinical strains of HIV, while maintaining equivalence in virus outgrowth kinetics. Importantly the use of *Gaussia* luciferase facilitates earlier detection of reactivated latent viruses and further streamlines the VOA.

## Results

### Construction of a novel indicator cell line, Sup-GGR

**Figure 1:**
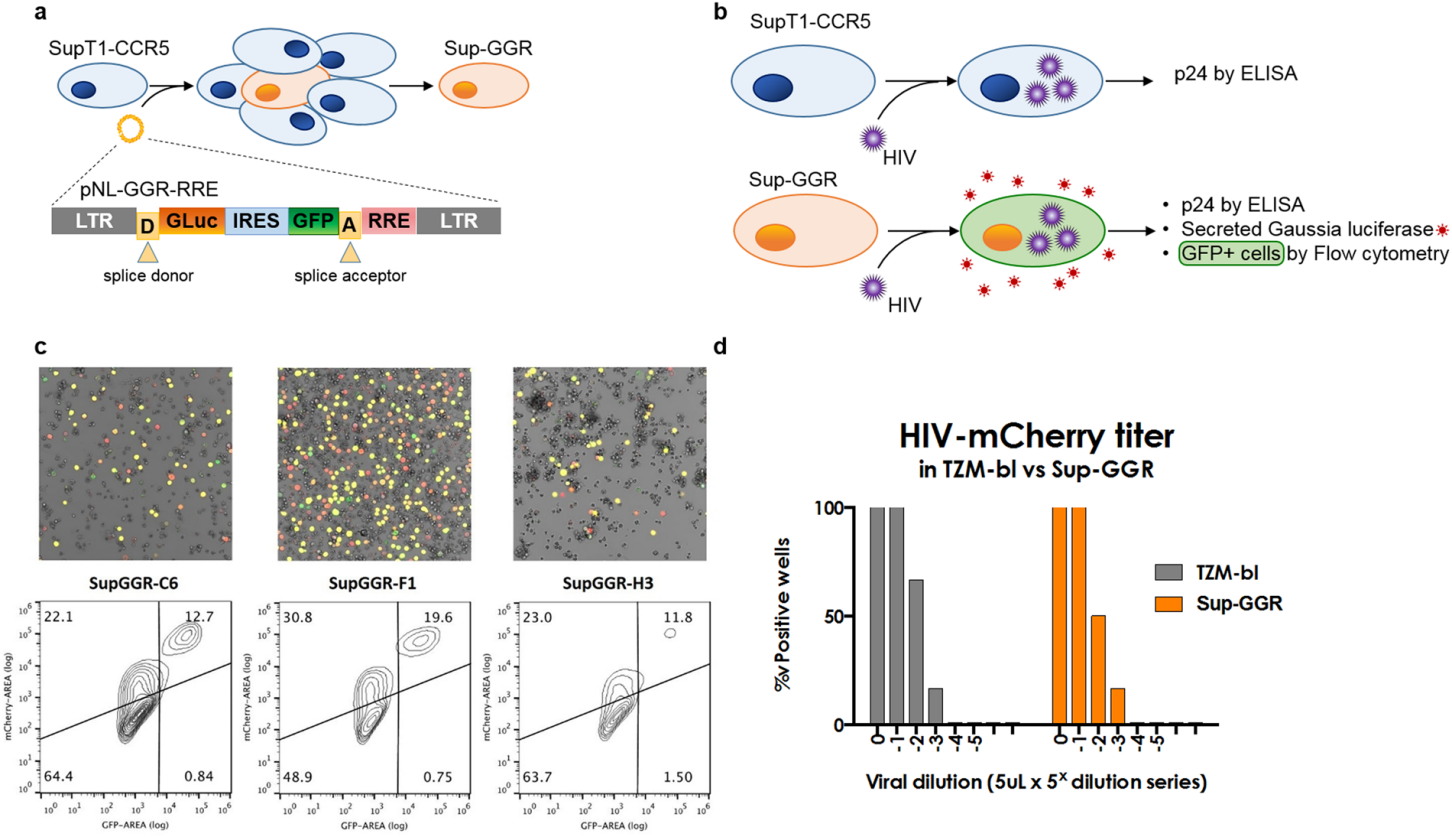
Construction of Sup-GGR cells. (**a**) Schematic of SupT1-GGR5 derivation. SupT1-CCR5 cells were transduced with the lentiviral reporter vector pNL-GGR-RRE (SA), and cloned by limiting dilution. The bicistronic expression cassette contains two reporter genes under the control of a Tat-dependent HIV LTR promoter. The incorporation of the HIV major splice donor and acceptor sites and a Rev Response Element also renders reporter expression Rev dependent. (**b**) Compared to the parental SupT1-CCR5 (top panel), Sup-GGR offers two additional readouts of HIV activity – GLuc and hrGFP expression. (**c**) Individual Sup-GGR subclones C6, F1 and H3 were infected with VSV/G-HIV-IRES-mCherry replication competent HIV, and the GGR reporter signal was assayed by fluorescence microscopy (top row) and flow cytometry (bottom) at six days post-infection. Clone F1 gave the highest proportion of dual positive cells (top center quadrant) with trivial background hrGFP expression and was chosen for further characterization. (**d**) Titer (TCID50/mL) for a stock of non-pseudotyped HIV-IRES-mCherry (BaL env) was calculated by serial dilution in Sup-GGR (orange, TCID50_Sup_ = 0.72 × 10^4^/mL) and the widely employed cell reagent, TZM-bl (grey, TCID50_TZM_ = 1.16 × 10^4^/mL), showing comparable susceptibility to HIV infection.

We modified SupT1-CCR5 T-lymphoblastic lymphoma cells, known to support the replication of both of X4 and R5 tropic HIV, to express two independent indicator genes upon viral infection, Gaussia luciferase (GLuc) and humanized *Renilla* GFP (hrGFP)^6^. We used a previously published Tat/Rev-dependent vector^5^, pNL-GGR-RRE (SA), to create the Sup-GGR (**G**aussia **G**FP **R**eporter) cell line. The bicistronic reporter cassette contains GLuc and hrGFP coding sequences separated by an internal ribosome entry site (IRES), and is flanked by HIV major splice donor and acceptor sequences. The reporter genes are transcribed under the control of the pNL4-3 HIV LTR promoter. The presence of the Rev-responsive element (RRE) placed downstream of the hrGFP reporter allows the specific transcription and translation of GLuc and hrGFP genes only in the presence of both Tat and Rev (Fig. 1a). With this reporter cassette, HIV infection can be detected by quantification of either the GLuc signal in the culture supernatant or hrGFP fluorescence by flow cytometry, microscopy or by plate reader, in addition to the conventional p24 ELISA assay (Fig. 1b).

The SupT1-CCR5 cell line was transduced with the pNL-GGR-RRE (SA) reporter lentiviral vector, expanded in culture and cloned by limiting dilution (Fig. 1). More than 200 subclones were obtained and were split and replated in replica plates, one replicate of each was infected with stocks of HIV-IRES-mCherry (BaL env), and screened for GFP fluorescence by confocal microscopy (data not shown). The HIV-IRES-mCherry vector is a full length replication-competent infectious clone based on NL4-3, but encodes the BaL envelope and carries an IRES-mCherry cassette downstream of Nef^7^. Of the forty-five clones that yielded a GFP-positive signal upon infection, three clones were selected by microscopy for infection penetrance and GFP reporter intensity: Sup-GGR subclones C6, F1 and H3.

To compare the ability of these 3 subclones to report HIV infection, hrGFP reporter signal intensity was assayed by fluorescence microscopy and flow cytometry 6 days post-infection with HIV-IRES-mCherry (BaL env), pseudotyped with VSV-G to enhance first round entry (Fig. 1c). All three Sup-GGR clones expressed hrGFP upon HIV infection but with variability between the clones. A higher proportion of hrGFP+/mCherry+ cells was seen in Sup-GGR-F1 compared to Sup-GGR-C6 and Sup-GGR-H3. The same profile was observed by flow cytometry with 19.6% of cells positive for both GFP and mCherry for Sup-GGR-F1 compared to 12.7% for Sup-GGR-C6 and 11.8% for Sup-GGR-H3. A proportion of cells were infected with mCherry virus but did not express hrGFP (30.8% for Sup-GGR-F1, 22.1% for Sup-GGR-C6 and 23% for Sup-GGR-H3); these mCherry(low) cells presumably correspond to early infection prior to induction of the hrGFP reporter. Importantly, double-positive hrGFP+/mCherry+ cells were 1.5–2 logs higher in mCherry fluorescence than single positive hrGFP-/mCherry(low) cells. Additionally a very small proportion of cells expressed hrGFP in the absence of HIV infection (0.75% for Sup-GGR-F1, 0.84% for Sup-GGR-C6 and 1.50% for Sup-GGR-H3). Based on these results, the subclone Sup-GGR-F1 was selected for subsequent experiments.

To characterize the Sup-GGR-F1 clone further, we infected it with serial dilutions of HIV-IRES-mCherry (BaL env) stocks, and calculated the 50% tissue culture infective dose (TCID50/mL) compared to TZM-bl cells (Fig. 1d). Both cell types demonstrated comparable sensitivity to HIV infection with a TCID50 of 0.72 × 10^4^/mL and 1.16 × 10^4^/mL for SupGGR-F1 and TZM-bl, respectively. The Sup-GGR-F1 clone was thus renamed Sup-GGR and selected for additional validation.

### Sensitivity of Sup-GGR cell line to infection by replication-competent laboratory HIV strains

**Figure 2:**
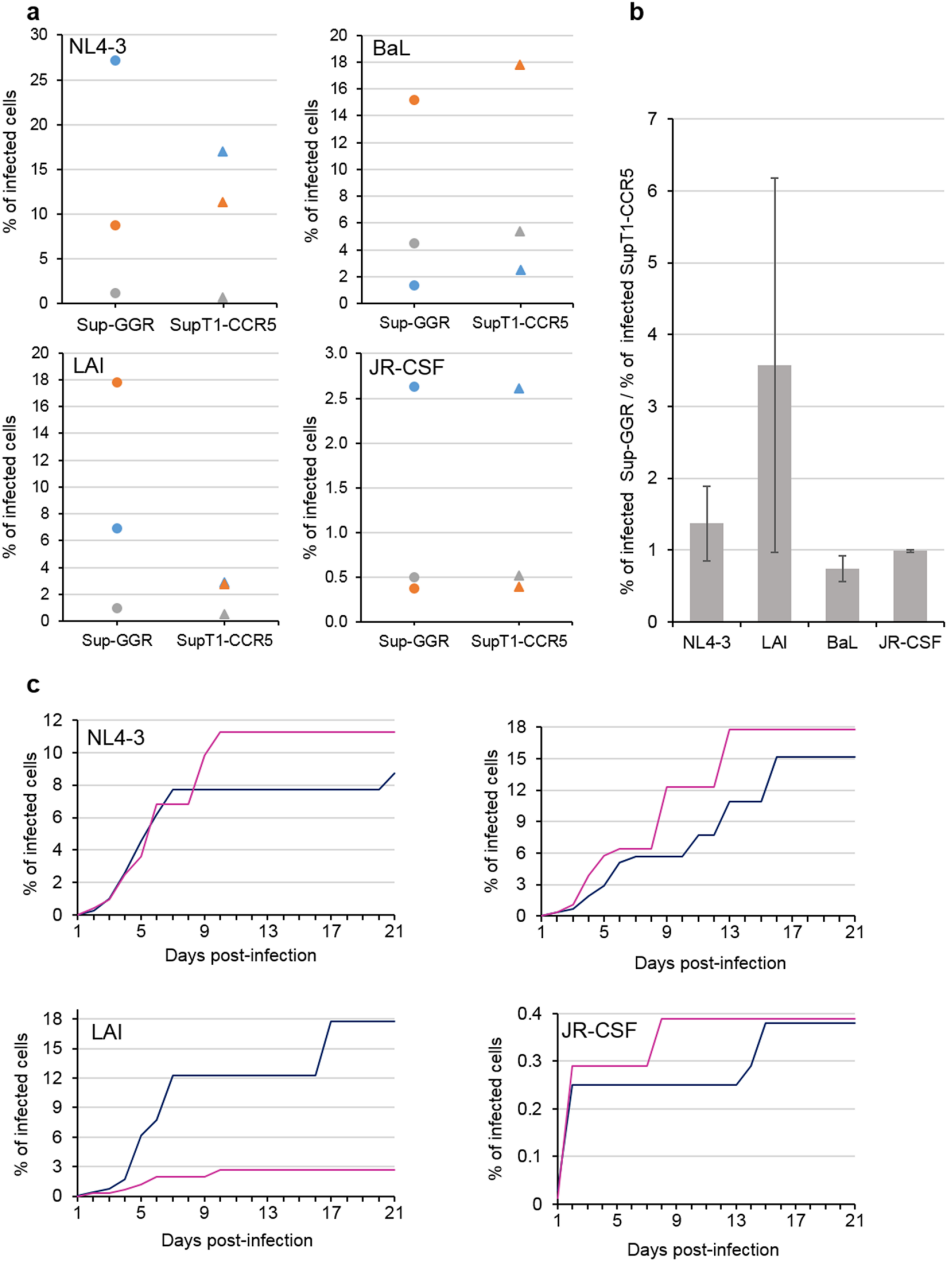
Sup-GGR can readily detect X4-tropic viruses (NL4-3 and LAI) or R5-tropic viruses (BaL and JR.CSF). (**a**) SupT1-CCR5 cells were infected with various strains of viruses (NL4-3, LAI, BaL or JR-CSF) and serial fivefold dilution (starting from 625 cells per well) of infected cells were co-cultured with uninfected Sup-GGR (circle) or SupT1-CCR5 (triangle) in 96 well plates, with 10 wells/dilution. Cytopathic effects were assessed every day for 21 days and the percentage of infected cells in the stock was calculated based on limiting dilution statistics. Three independent experiments are represented, each experiment is depicted by a different color (blue, grey or orange). (**b**) Ratio of the percentage of infected cells in the stock detected by Sup-GGR over that detected by SupT1-CCR5 for each strain of virus, using data from (**a**). Three independent experiments were performed. Error bars indicates standard deviation. The ratios are around 1 for all four strains of viruses indicating that SupGGR is at least as efficient as SupT1-CCR5 in supporting HIV replication. (**c**) Percentage of infected cells as determined for Sup-GGR (blue) or SupT1-CCR5 (pink) at different days post-infection demonstrating the kinetics of infection. One representative experiment from 3 replicates is presented.

We tested the ability of Sup-GGR cells to capture and sustain replication of X4 and R5 tropic HIV viruses in a cell-to-cell transfer milieu compared to the parental SupT1-CCR5 line. SupT1-CCR5 cells were infected with X4 tropic viruses, NL4-3 and LAI, or R5 tropic viruses, BaL and JR-CSF. HIV infected SupT1-CCR5 cells were used as donor cells and then co-cultured at serial fivefold dilutions with either uninfected SupT1-CCR5 or uninfected Sup-GGR. Each dilution was assayed in 10 replicates and cytopathic effects (CPE) were checked every day for 21 days. The percentage of infected cells was determined by limiting dilution statistics^8^. Figure 2a shows the results of three independent experiments. The percentage of infected cells in each experiment was variable, but crucially the results obtained from Sup-GGR were comparable to that from SupT1-CCR5 irrespective of the input donor cell number for three (NL4-3, BaL and JR-CSF) out of four strains of viruses. This is also illustrated in Fig. 2b showing the ratio of the percentage of infected cells detected by Sup-GGR over that detected by SupT1-CCR5 for each virus strain. A ratio >1 would indicate Sup-GGR was better able to support the replication of the strain of virus tested compared with SupT1-CCR5, and a ratio <1 would indicate Sup-GRR was less able to support the replication of this strain of virus. As the ratios for NL4-3, BaL and JR-CSF are around 1 (1.37, 0.73 and 0.98 for NL4-3, BaL and JR-CSF, respectively), Sup-GGR are demonstrably as efficient as Sup-CCR5 in supporting the replication of these strains of virus (Fig. 2b). For LAI, the ratio is 3.57 but the 95% confidence limits of the ratio is between 0.62 and 6.53 and is not statistically significantly greater than 1 (Fig. 2a,b). Representative kinetics from three replicate experiments are shown in Fig. 2c. The Sup-GGR cells exhibit similar kinetics of infection compared with SupT1-CCR5 for all X4 and R5 strains tested. Thus Sup-GGR cells support HIV replication of both X4 and R5-tropic laboratory strains at least as efficiently as SupT1-CCR5 cells line and with similar kinetics.

### Efficient detection of reactivated latent viruses from CD4+ T cells isolated from HIV-1 infected individuals by Sup-GGR cell line

Virus outgrowth assay currently provides the most definitive minimal estimate of the size of the latent reservoir^9^. The VOA involves limiting dilution of patient-derived resting CD4+ T cells, followed by reactivation of latent viruses *in vitro*. Reactivated latent viruses are allowed to replicate in reporter cells both to demonstrate that the virus is replication competent and to allow signals to amplify for accurate quantitation. The culture is sampled at different time-points for evidence of viral activity. We previously reported that using SupT1-CCR5 as reporter cells to facilitate the replication of reactivated latent viruses in VOA improves the reproducibility of the assay compared to using uninfected donor-derived T-lymphocytes^6^. Here we assessed the capacity of Sup-GGR cells to detect reactivated latent viruses from primary resting CD4+ T cells from HIV infected patients. Briefly, resting CD4+ T cells were purified from patients and were subjected to limiting dilution such that approximately a third to a half of all wells are predicted to contain an inducible latent virus. Resting cells were activated with phytohaemagglutinin-L (PHA-L) and irradiated allogeneic PBMCs in the presence of IL-2, and co-cultured with either Sup-GGR or SupT1-CCR5. In addition wells containing Sup-GGR without resting CD4+ T cells were used as a negative control for baseline GLuc and hrGFP expression. Samples were harvested at regular intervals for p24 ELISA, luciferase and flow cytometry analyses. Supplementary Figure S1 illustrates the threshold applied for negative vs positive wells for flow cytometry and GLuc detection.

**Figure 3:**
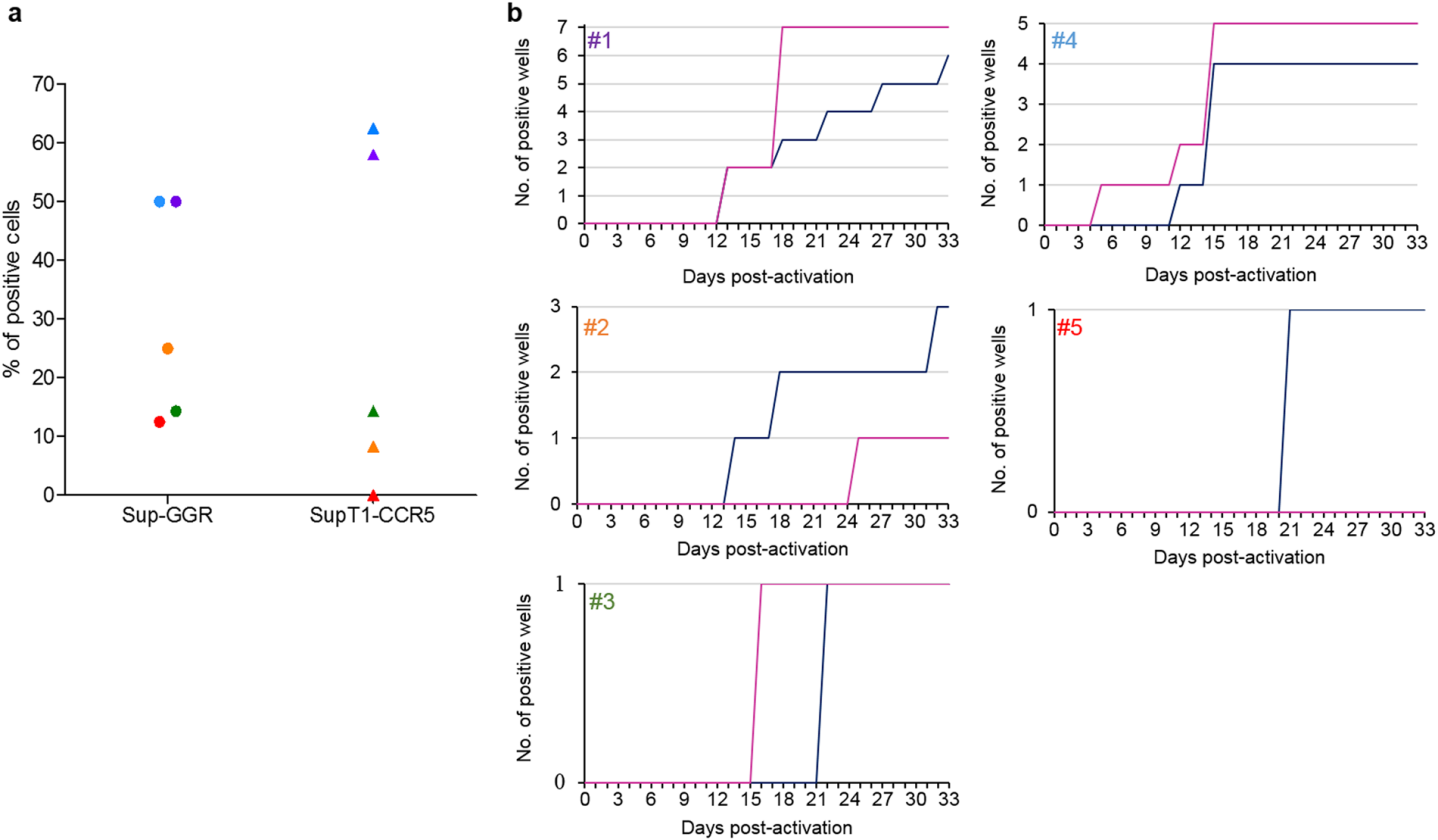
SupT1-GGR5 cells are as sensitive as SupT1-CCR5 in co-culture assay. Highly purified resting CD4+ T cells were obtained from whole blood of five HIV+ patients. These cells were activated with PHA-L and allogeneic irradiated PBMC, seeded at 4–8 × 10^5^ cells per well (depending on donor), and co-cultured with either SupT1-CCR5 or Sup-GGR. Samples were harvested regularly for p24 ELISA, GLuc detection and flow cytometry analyses. (**a**) Percentage of positive wells from five different patients when stimulated resting CD4+ T cells were co-cultured with each cell type, using p24 ELISA as readout. Each color represents resting CD4+ T cells isolated from one patient co-cultured with either Sup-GGR (circle) or SupT1-CCR5 (triangle). The total number of wells seeded for each patient is shown in Table 1. There is no significant difference between Sup-GGR and SupT1-CCR5 (p = 0.75, Wilcoxon matched-pairs signed rank test, two tailed test). (**b**) Virus outgrowth kinetics using Sup-GGR cell line (blue) or SupT1-CCR5 (pink). Results represent *ex vivo* co-culture from five different patients (#1 to #5).

We first compared the percentage of positive wells detected by p24 ELISA in either Sup-GGR or SupT1-CCR5 co-culture wells at 33 days post-activation. The analysis was performed on five HIV+ patients (Supplementary Table S1). As shown in Fig. 3a, within each patient the proportion of positive wells co-cultured with SupT1-CCR5 and Sup-GGR were similar (50% vs 58%, 25% vs 8.3%, 14.3% vs 14.3%, 50% vs 62.5% and 12.5% vs 0 for patient #1 to #5, respectively). Furthermore there is no significant difference between Sup-GGR and SupT1-CCR5 (p = 0.75, Wilcoxon matched-pairs signed rank test, two tailed test). Table 1 summarizes the number of positive wells obtained for each patient in either in Sup-GGR and SupT1-CCR5. Similarly, the kinetics of virus outgrowth, whilst variable between patients, were similar in Sup-GGR and SupT1-CCR5 for each patient (Fig. 3b). Overall these experiments showed that the Sup-GGR cell line is at least as efficient as SupT1-CCR5 in supporting replication of HIV reactivated *ex vivo* from primary CD4+ T cells.

**Table 1:** Number of positive wells detected in Sup-GGR vs SupT1-CCR in VOA.

	Sup-GGR	SupT1-CCR5
#1	6/12	7/12
#2	3/12	1/12
#3	1/7*	1/7
#4	4/8	5/8
#5	1/8*	0/8

The number of wells used for each patient is dependent on the number of resting cells available from each venesection. Asterisks identify patients for which one additional well was found positive with luciferase in Sup-GGR but not with ELISA. There is no significant difference between Sup-GGR and SupT1-CCR5 (p = 0.75, Wilcoxon matched-pairs signed rank test, two tailed test).

**Figure 4:**
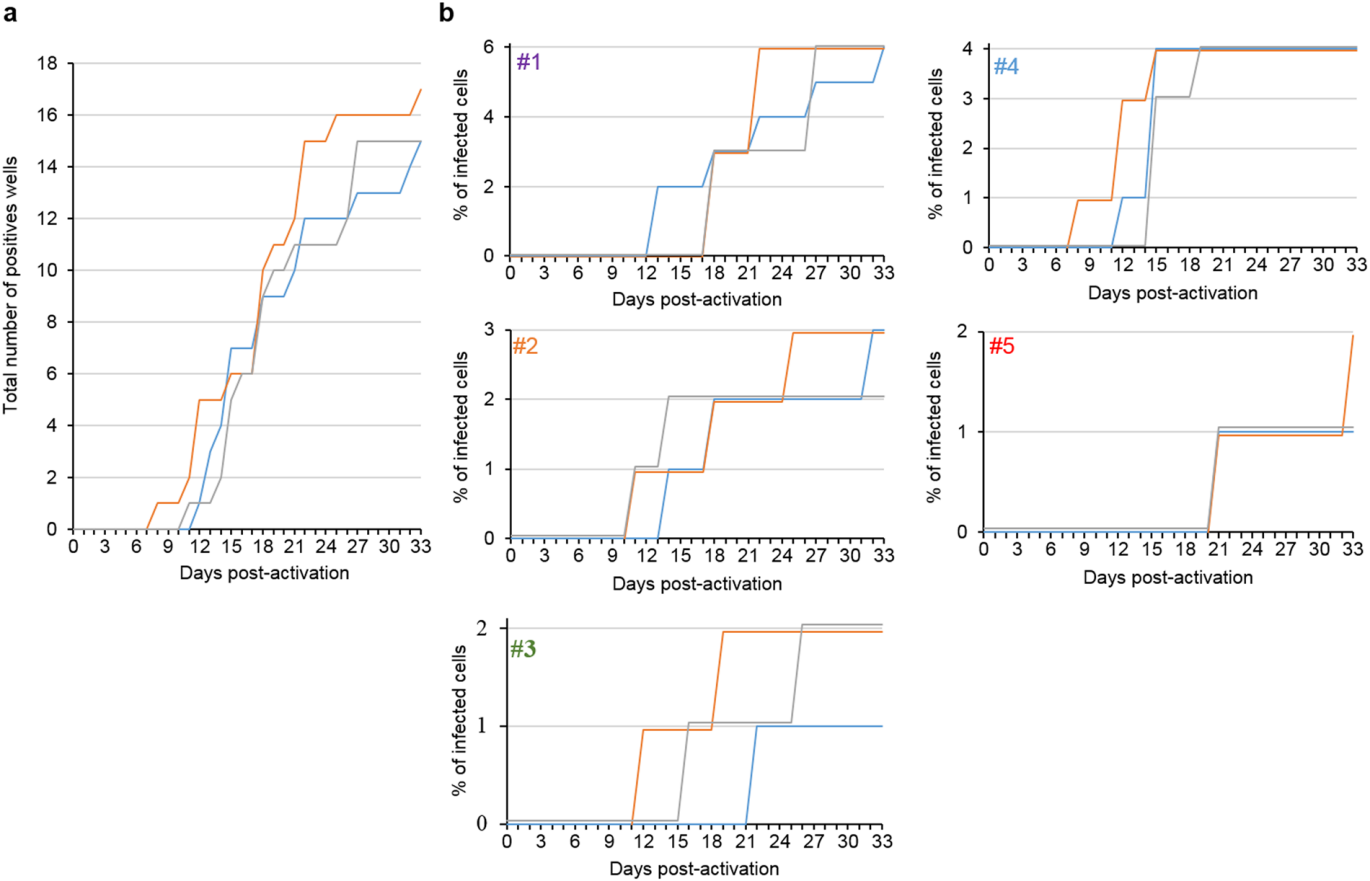
Luciferase facilitates earlier detection of reactivated latent viruses compared to p24 ELISA and flow cytometry. (**a**) Total number of positive wells aggregated from all five patients, as determined by p24 ELISA (blue), luciferase (orange) and flow cytometry (grey) at different days of co-culture. (**b**) Virus outgrowth kinetics as determined by p24 ELISA (blue), luciferase (orange) and flow cytometry (grey) for each patient (#1 to #5).

Sup-GGR cells express GLuc and hrGFP when infected with HIV. We analysed both readouts in addition to p24 ELISA during VOA and compared their efficiency in detecting reactivated viruses. Figure 4a, showing the cumulative total number of positive wells from all five patients over the course of VOA, illustrates the kinetics of virus outgrowth as determined by the three readouts. Table 2 shows the day at which a well becomes positive with various modes of detection. The table contains data from 17 positive wells derived from 5 patients. In 11/17 samples GLuc gave an earlier detection, in 4/17 they were equivalent, and only in 2/17 did conventional p24 ELISA yield an earlier detection. The average time to detection is shorter with GLuc at 17.8 days (median 18 days) post-activation, compared with 21.5 days (median 18 days) with p24 ELISA and 20.8 days (median 19 days) with flow cytometry for hrGFP (GLuc vs p24 ELISA, p = 0.03; GLuc vs flow cytometry, p = 0.001, two tailed ‘t’ test). This translates to a significant reduction in time to culture-positivity requirement for VOA (Table 3). In the five patients tested, to capture all the positive wells, on average 22.8 days (median 22 days) would be required with GLuc as readout, compared to 27.6 days (median 27 days) for flow cytometry and 31 days (median 33 days) for p24 ELISA (GLuc vs p24 ELISA, p = 0.04; GLuc vs hrGFP, p = 0.03, two tailed ‘t’ test). In addition to detecting positive wells sooner, we unexpectedly observed that in two wells (one from patient #3 and one from patient #5), viral activity could be detected by GLuc but not by p24 ELISA. We could observe CPE in these wells and one of them was also positive by flow cytometry (patient #3) (Fig. 4b).

**Table 2:** Time to detection for the positive wells (days post activation) by the mode of readout for all five patients in VOA (#1 to #5).

	Well	GLuc	p24	hrGFP
#1	1	18	13	18
	4	22	33	27
	5	18	13	18
	7	22	27	27
	11	22	22	27
	12	18	18	18
#2	2	11	14	11
	3	14	18	14
	10	25	32	35*
#3	1	12	36*	19
	2	19	22	22
#4	1	12	15	15
	2	12	15	15
	4	15	15	19
	6	8	12	15
#5	4	21	21	21
	6	33	39*	33
average		17.8	21.5	20.8
median		18	18	19

Asterisks indicate the last censored day rather than day when the readout was positive. The average time to detection is shorter with *Gaussia* luciferase compared with both p24 ELISA and flow cytometry (GLuc vs p24 ELISA, p = 0.03; GLuc vs flow cytometry, p = 0.001, two tailed ‘t’ test).

**Table 3:** Times for all wells to turn positive in VOA for each patient by the mode of detection.

	GLuc	p24	hrGFP
#1	22	33	27
#2	25	32	35*
#3	19	36*	26
#4	15	15	19
#5	33	39*	33
average	22.8	31	27.6
median	22	33	27

Asterisks indicate last censored date when a well tested positive by another method remains negative with the indicated mode of detection (GLuc vs p24 ELISA, p = 0.04; GLuc vs hrGFP, p = 0.03, two tailed ‘t’ test).

Altogether these results showed that the new indicator cell line, Sup-GGR, is at least as efficient as SupT1-CCR5 in supporting replication of HIV reactivated *ex vivo* from primary resting CD4+ T cells. In addition, *Gaussia* luciferase assay facilitates earlier detection of viral outgrowth. As the detection of GLuc secreted by Sup-GGR can be achieved without lysis of the cells, the indicator cell is an excellent tool to detect reactivated virus in a VOA setting.

## Discussion

Indicator cell lines that can detect the presence of virus are essential tools in virological research. Here we describe a new dual-indicator cell line, Sup-GGR, that expresses GLuc and hrGFP upon HIV infection. Systems such as HIV latency cell lines^10,11^, and cells containing bicistronic promoter/reporter constructs^12–18^ have been previously described and are valuable tools to study HIV. These can allow identification and/or isolation of cells containing an integrated provirus^12^, study of LTR function^16^, and screening for antiviral activity^13,15^. Unlike prior systems however, our dual-indicator cell line is a reporter system primarily designed to detect the presence of replication competent HIV reactivated from primary CD4+ T cells. Existing indicator cell lines for HIV-1^2^ must be lysed to allow reporter gene expression readout, and thus cannot be used for repeat sampling of an individual culture. Importantly, GLuc detection allows extended follow-up of HIV replication because it is secreted into the supernatant obviating cell lysis. Sup-GGR cells support the replication of both X4- and R5-tropic HIV, at least as efficiently as the parental SupT1-CCR5 line. We did not observe any discrepancy in the detection of HIV with this reporter with the five clinical isolates compared to conventional p24 assay. Here we have not formally tested the reporter on all the subtypes of HIV. The reporter genes rely on the activity of viral Tat and Rev, and genetic and functional variations in Tat^19–21^, and Rev^22^ between different strains of viruses has been described. Similar to the current HIV-1 LTR-based reporter cells available in the field, there is thus a theoretical concern that the reporter cassette may report infection with some viral strains at a lower efficiency, and indeed may be incompatible with others. Our ongoing pursuits employ Sup-GGR cells to study the kinetics and biology of full-length HIV isolates, including uncloned swarms from patient material; such experiments are traditionally pursued using cloned envelopes in reporter lentiviral vectors and can only study envelope-mediated viral entry.

We demonstrated the utility of Sup-GGR in detecting reactivated latent viruses. Sup-GGR has equivalent sensitivity when deployed in VOA, compared with the parental SupT1-CCR5 cell line. Virus outgrowth kinetics are similar in a head to head comparison with SupT1-CCR5, using p24 ELISA as readout. We have also compared the three readouts of viral activity - supernatant p24, supernatant *Gaussia* luciferase and hrGFP, available with Sup-GGR in VOA. In this context, we did not find flow cytometry for hrGFP expression offered significant advantages over supernatant p24 ELISA. HIV infection is lethal to Sup-GGR, thus limiting the window during which infected cells are both viable and hrGFP positive to be included in analysis. In addition, the VOA protocol requires Sup-GGR to be co-cultured with irradiated allogeneic PBMC and *ex vivo* CD4+ T cells. There is thus a large background of cells that will not express GFP, limiting the sensitivity of this readout.

Interestingly we observed two wells (patient #3, well 1, patient #5, well 6) where Tat/Rev reporter driven readouts (*Gaussia* luciferase) were positive with Sup-GGR, and supernatant p24 ELISAs were negative. This may represent very early stages of viral replication prior to sufficient accumulation of budded viruses in the supernatant to allow detection by p24 ELISA assay. Importantly, tools such as Sup-GGR afford the opportunity to study such events that may be missed using standard platforms for viral outgrowth quantitation.

One key improvement with Sup-GGR is the significant advantages that secreted *Gaussia* luciferase provide over conventional p24 ELISA both in terms of detection and labor. Whilst there is patient-to-patient variation in the latent load (which is expected), in most cases positive wells can be identified by *Gaussia* luciferase significantly earlier than by p24. This is true irrespective of the manner of analysis: by the proportion of positive wells detected by various methods, by the average time to positivity, and most importantly for use in VOA, by the overall time required to capture all positive wells. For this study we prolonged the tissue culture period in VOA to 33 days instead of the usual 23 days. We found that just under one third of all wells which eventually turned positive did so late and would have been scored as negative with the standard VOA protocol where viruses were cultured for three weeks and ELISA was used as the readout. Thus premature termination of VOA may also lead to an underestimation of the size of the latent reservoir. However, more than half of those wells would be scored as positive if luciferase were used as the readout, without the need to prolong the culture period. In addition, using *Gaussia* luciferase as readout also offered significant time savings with respect to readout. In our hands *Gaussia* luciferase could be read in under half an hour, whilst an ELISA requires nearly 5 hours of laboratory time. Such savings can improve the scalability of VOA.

In summary we report here the construction and validation of a new dual-indicator cell line, Sup-GGR, to assess HIV replication. Sup-GGR expresses two readout signals when infected with HIV, *Gaussia* Luciferase which is secreted into the supernatant and intracellular hrGFP. Unlike other indicator cells, Sup-GGR permits repeated samplings without terminating the culture. Sup-GGR can potentially be used in a wide range of applications, such as in extended experiments like VOA to track HIV replication.

## Materials and Methods

### Patient cohort

The participants gave written informed consent and this study was approved by the National Health Services (NHS) Health Research Authority (UK) under REC reference 12/SC/0679. All experimental procedures were approved by the institutional review boards of the University of Cambridge and were performed in accordance with the relevant guidelines. Clinical characteristics of the patients are described in Supplementary Table 1.

### Cells

TZM-bl cells were obtained through the NIH AIDS Reagent Program, Division of AIDS, NIAID, NIH: TZM-bl from Dr. John C. Kappes, Dr. Xiaoyun Wu and Tranzyme Inc. SupT1-CCR5 cells were a generous gift from Dr. James Hoxie, University of Pennsylvania. Affinofile-GGR cells were graciously provided by Dr. Benhur Lee, Mount Sinai School of Medicine. 293FT cells were purchased from Invitrogen.

### Viruses

HIV clones JR-CSF, BaL, NL4-3, and LAI were acquired through the NIH AIDS Reagent Program. HIV-IRES-GFP (BaL env) was a gift of Drs. Thorsten Mempel, MGH, and Thomas Murooka, University of Manitoba. HIV-IRES-mCherry (BaL env) was generated by cloning the mCherry cDNA into HIV-nef-IRES-GFP, using PCR amplification from pCAAGS-mCherry (a kind gift of Dr. Natasza Kurpios, Cornell University) and cloning a 1.7 kb MluI-IRES-mCherry-LTR-*Xba*I cassette into digested pNL43-IRES-GFP(BaL env).

### Cell culture

SupT1-CCR5 cells and Sup-GGR were maintained in RPMI 1640 with L-glutamine supplemented with 10% Fetal Bovine Serum and 1% penicillin/streptomycin. SupT1-CCR5 and Sup-GGR cells were split 1:10 twice weekly. Adherent cell lines TZM-bl, Affinofile-GGR, and 293-FT were maintained in DMEM with L-glutamine supplemented with 10% Fetal Bovine Serum, 1X sodium pyruvate, 1X HEPES and 1% penicillin/streptomycin. Cells were split 1:10 every three days.

### Fluorescence microscopy

Fluorescent signal from reporter cells or infectious HIV clones encoding fluorophores were imaged live under BSL3 containment on a Leica SP-5 laser scanning confocal microscope using either a universal plate holder for glass-bottom or chamber glass culture formats, or the H201–MEC–LG–MW holder (OkoLabs) for optical bottom 96-well plate culture assays. Z-stacks were projected, and channels merged and contrasted in Leica Application Suite or Adobe Photoshop.

### Establishment of an indicator cell line carrying the Gaussia luciferase and GFP signal

Affinofile-GGR cells were transfected with pCMV-dR8.2-dvpr and pLP-VSV/G to package the GGR provirus integrated in these cells. At 48-hours post-transfection, harvested supernatant was used to spinoculate target SupT1-CCR5 cells at 1000 × g for two hours at room temperature in the presence of 10 µg/mL DEAE-dextran. Infected cells were then resuspended in fresh medium, expanded for one week in culture, and plated at limiting dilution (~0.5 cells/well) in 96-well round bottom dishes. Plates were spun at 2000 × *g* for 30 minutes, and single cells verified by phase contrast microscopy. Cell clones were expanded for two weeks, then split among four replicate plates and further expanded for testing of reporter activity.

### Reporter screening in Sup-GGR cell clones

Subclones of Sup-GGR were screened for reporter activity by infection with VSV-G pseudotyped or wild type full length HIV-IRES-mCherry (BaL env) HIV stocks. Wells were live imaged by confocal microscopy, and reporter fluorescence was quantified either live on a BioRad S3e cell sorter or following fixation (BD Cytofix/Cytoperm) on a BD LSRII flow cytometer.

### Infection of SupT1-CCR5 and Sup-GGR with laboratory virus strains

SupT1-CCR5 were infected with different strains of viruses: NL4-3, LAI, BaL or JR-CSF. Cells were carefully washed three times to remove excess virions. Serial fivefold dilution of these stocks of infected cells were co-cultured with 50000 cells/well of either uninfected Sup-GGR or SupT1-CCR5 in 96 well plates with ten wells for each dilution. Wells were checked for cytopathic effects every day for 21 days and the percentage of infected cells in the stock was calculated based on limiting dilution statistics^8^. Three independent experiments were performed.

### 50% tissue culture infective dose (TCID50)

Stocks of HIV-IRES-mCherry were titered on TZM-bl and Sup-GGR cells. To 2,500 target cells in 96-well format were added 5 μL of five-fold serially-diluted HIV stock, in the absence of DEAE-dextran. At 72 hours post-infection, HIV-mCherry positive wells were scored and the fraction of positive wells used to discern the TCID50/mL for each viral stock according to the Reed-Muench method^23^.

### Quantitative viral outgrowth assay

VOA was performed as previously described with modifications^6^. Briefly, PBMCs were isolated from whole blood by density gradient centrifugation using Lymphoprep (Alere Technologies AS, Oslo, Norway). Resting (CD25^−^, CD69^−^, HLA-DR^−^) CD4+ T cells were negatively selected using a custom antibody cocktail (StemCell Technologies) and cultured with 20 nM efavirenz and 100 nM raltegravir for 1 to 2 days to allow for the degradation of unintegrated viral DNA. On ‘day 1’ of the assay, cells were washed thoroughly to remove antiretroviral drugs, stimulated with 10-fold excess of irradiated allogeneic PBMCs and 2 μg/ml PHA-L in RPMI medium containing 10 U/ml IL-2, and seeded at 4–8 × 10^5^ resting cells per well in 12 well plates. 24 hours after stimulation, media containing PHA-L was removed and replaced with 3 ml of fresh media containing IL-2 and SupT1-CCR5 or Sup-GGR cells at 5 × 10^5^ cells per well. Negative control wells containing Sup-GGR without resting CD4+ T cells were included to account for background luciferase and hrPGF expressions in subsequent analyses. After the first week, half of the media and cells were harvested twice weekly and replaced with fresh media with IL-2. The culture was maintained for a minimum of 33 days. Supernatant was inactivated in 0.1% Empigen at 56 °C for 30 minutes and analysed for HIV-1 p24 production by ELISA and *Gaussia* luciferase expression (Pierce *Gaussia* Luciferase Flash Assay Kit, Thermo Scientific). Cell were washed twice, fixed in 300 µL of 4% paraformaldehyde and GFP+ cells were quantified by flow cytometry (BD Accuri C6). Supplementary Figure S1 illustrates the threshold applied for negative vs positive wells for flow cytometry and GLuc detection.

## Supplementary information

Supplementary information
