# Supplementary material for: A novel, sensitive dual-indicator cell line for detection and quantification of inducible, replication-competent latent HIV-1 from reservoir cells
Source: Sci Rep. 2019 Dec 18;9:19325. doi: 10.1038/s41598-019-55596-8 (PMC6920355; doi:10.1038/s41598-019-55596-8)
Supplement: Supplementary file 1 — Supplementary information [file 41598_2019_55596_MOESM1_ESM.pdf]

A novel, sensitive dual-indicator cell line for detection and quantification of inducible, replication-competent latent HIV-1 from reservoir cells

Fanny Salasc[1#]

David W. Gludish[2#],

Isobel Jarvis[1],

Saikat Boliar[2],

Mark R Wills[1],

David G. Russell[2*]

Andrew ML Lever[1*],

Hoi-Ping Mok[1*]

[#] Both authors contributed equally to the work

Affiliations: [1] Department of Medicine, University of Cambridge, Cambridge, UK, [2] Cornell University College of Veterinary Medicine, New York, USA

Supplementary Table S1

	age	gender	months from last VL>400	months from last VL>50
**#1**	63	M	95	41
**#2**	46	M	>42	>42
**#3**	52	F	>92	>92
**#4**	48	M	66	3
**#5**	48	M	115	20

Clinical characteristics of patients whose blood was used in VOA

Supplementary Figure S1

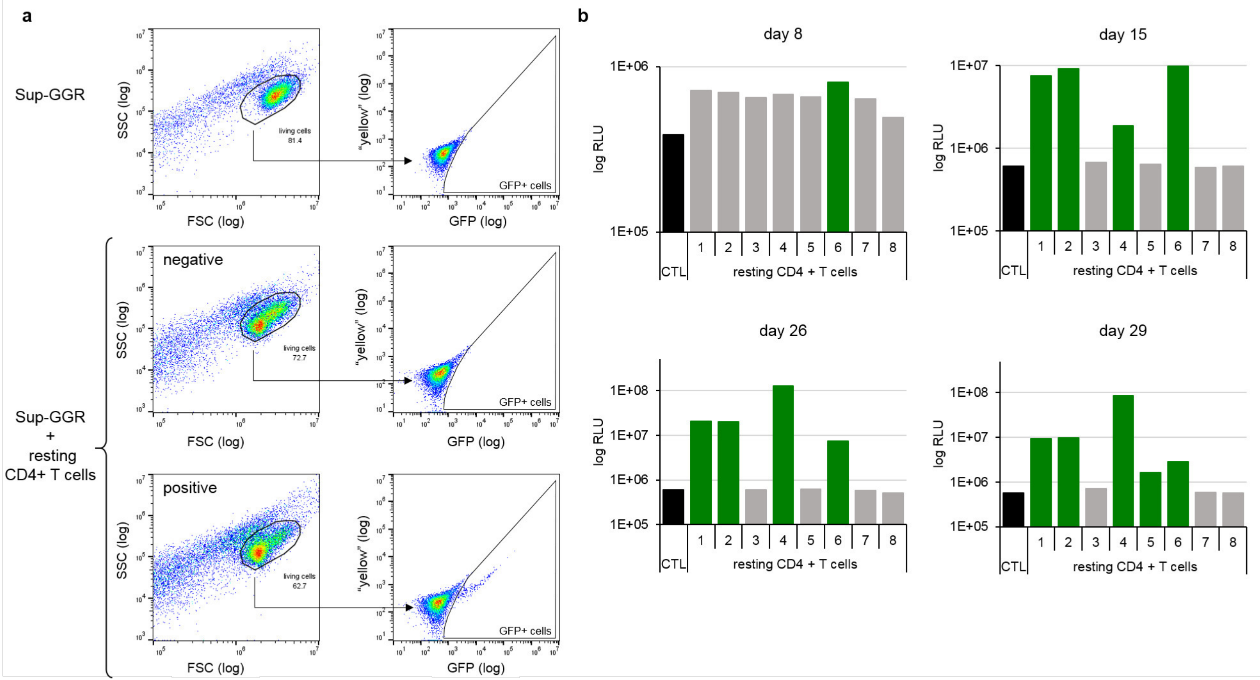

Parameters for analysis of GLuc detection and flow cytometry in VOA assay. All data presented are from patient #4 (a) GFP+ cells were quantified by flow cytometry. We first selected the live cell population (left panel, gated cells) and then compared the GFP signal (gated in right panel) obtained for negative control (Sup-GGR cells, upper panel) to those of VOA samples (Sup-GGR cells cultivated with resting CD4+T cells from seropositive donor, middle and bottom panels). Shown is an example of a negative sample (middle panel) and a positive sample (bottom panel). GFP+ cells are identified on the X-axis. To distinguish positive cells from autofluorescence we used 585/40 emission filter on the Y-axis. (b) Gaussia luciferase was measured over time in the supernatant of Sup-GGR (CTL, in black) or Sup-GGR with resting CD4+T cells of seropositive donors (wells 1-8 for each graph, negative well in grey, positive well in green). A well is considered positive if the RLU (relative luciferase unit) is 1/2 log higher than the control. Shown is an example of the kinetics for one experiment.
